# Evidence that a Panel of Neurodegeneration Biomarkers Predicts Vasospasm, Infarction, and Outcome in Aneurysmal Subarachnoid Hemorrhage

**DOI:** 10.1371/journal.pone.0028938

**Published:** 2011-12-09

**Authors:** Robert Siman, Nicholas Giovannone, Nikhil Toraskar, Suzanne Frangos, Sherman C. Stein, Joshua M. Levine, Monisha A. Kumar

**Affiliations:** 1 Department of Neurosurgery, School of Medicine, University of Pennsylvania, Philadelphia, Pennsylvania, United States of America; 2 Department of Neurology, School of Medicine, University of Pennsylvania, Philadelphia, Pennsylvania, United States of America; 3 Anesthesiology and Critical Care, School of Medicine, University of Pennsylvania, Philadelphia, Pennsylvania, United States of America; University of Cambridge, United Kingdom

## Abstract

Biomarkers for neurodegeneration could be early prognostic measures of brain damage and dysfunction in aneurysmal subarachnoid hemorrhage (aSAH) with clinical and medical applications. Recently, we developed a new panel of neurodegeneration biomarkers, and report here on their relationships with pathophysiological complications and outcomes following severe aSAH. Fourteen patients provided serial cerebrospinal fluid samples for up to 10 days and were evaluated by ultrasonography, angiography, magnetic resonance imaging, and clinical examination. Functional outcomes were assessed at hospital discharge and 6–9 months thereafter. Eight biomarkers for acute brain damage were quantified: calpain-derived α-spectrin N- and C-terminal fragments (CCSntf and CCSctf), hypophosphorylated neurofilament H,

14-3-3 β and ζ, ubiquitin C-terminal hydrolase L1, neuron-specific enolase, and S100β. All 8 biomarkers rose up to 100-fold in a subset of patients. Better than any single biomarker, a set of 6 correlated significantly with cerebral vasospasm, brain infarction, and poor outcome. Furthermore, CSF levels of 14-3-3β, CCSntf, and NSE were early predictors of subsequent moderate-to-severe vasospasm. These data provide evidence that a panel of neurodegeneration biomarkers may predict lasting brain dysfunction and the pathophysiological processes that lead to it following aSAH. The panel may be valuable as surrogate endpoints for controlled clinical evaluation of treatment interventions and for guiding aSAH patient care.

## Introduction

Subarachnoid hemorrhage (SAH), a severe form of hemorrhagic stroke caused by rupture of a brain aneurysm, affects 30,000 people annually in the United States [1], [2]. Of the 85% of patients who survive the initial aneurysmal SAH, approximately one-third develop further brain injury [2]. The majority of morbidity and mortality after aSAH is due to delayed cerebral ischemia (DCI) [3]. The cause of DCI has traditionally been ascribed to cerebral vasospasm, a narrowing of the cerebral arteries. However, therapies aimed at ameliorating vasospasm are only partially effective at preventing DCI [4]. DCI is defined clinically as focal neurological impairment or deterioration in neurological function on the Glasgow Coma Scale [5], and is correlated with radiographic evidence of cerebral infarction [6]. DCI typically occurs between 4–14 days after aSAH. Currently, there are few effective therapies for the prevention and treatment of DCI.

Biochemical markers for neurodegeneration of aSAH could increase the speed and discriminative power and reduce the cost of clinical research by serving as surrogate endpoints for experimental or non-optimized therapies. Such markers might also guide patient care through early detection of complications leading to DCI and long-term brain dysfunction. A number of proteins normally expressed predominantly in the nervous system increase markedly in cerebrospinal fluid (CSF) during the acute period following aneurysm rupture, and have been associated with increased patient mortality, morbidity and long-term brain dysfunction [7]–[12]. However, owing to one or more limitations in sensitivity, specificity, and reliability, no individual surrogate marker is widely accepted to predict vasospasm, DCI, or patient outcomes, or otherwise guide patient care [13]. To overcome these obstacles, we recently identified several new candidate protein markers for neurodegeneration and developed a novel biomarker panel for acute brain damage. Among the most abundant proteins released by degenerating neurons, several expressed predominantly or exclusively in the nervous system rise markedly in the CSF and blood of experimental animals subjected to ischemia- or trauma-induced acute brain damage [14], in some cases proportional to the severity of acute brain histopathology [15]. Moreover, several of the markers rise in the CSF and blood of human patients following surgically-induced circulatory arrest or traumatic brain injury, suggesting they might serve broadly in humans as biochemical indices of brain damage [16]–[19]. At least two, calpain-derived N- and C-terminal proteolytic fragments of the α-subunit of the actin-binding protein spectrin, are mechanism-based markers for the calpain-driven necrotic mode of neurodegeneration [20], [21] that is a major contributor to ischemic brain damage [22]–[24]. Here, we examined the prognostic utility of this panel of neurodegeneration biomarkers in severe aSAH in conjunction with widely studied neuronal and astroglial markers of acute brain damage, neuron-specific enolase and S100β, respectively. The kinetics and magnitude of CSF neurodegeneration biomarker alterations for up to 10 days after aneurysm rupture were compared with angiographic and ultrasonographic evidence for cerebral vasospasm, with clinical and neuroradiological evidence of brain ischemia and infarction, and with the degree of brain dysfunction at hospital discharge and long-term follow-up.

## Materials and Methods

### Ethics Statement

The study protocol was approved by the University of Pennsylvania Institutional Review Board (protocol #803321). All patients or their surrogates provided written informed consent. In cases where the patient was unconscious or cognitively impaired, written informed consent was obtained from the subject's authorized representative. All patient information was kept confidential in accordance with the Health Insurance Portability and Accountability Act.

### Aneurysmal subarachnoid hemorrhage patients

Twenty-five patients were recruited between 2007 and 2010. Inclusion criteria included: age between 18 and 80, Fisher group 3 or 4, and the presence of a saccular aneurysm confirmed by cerebral angiography. Patients presenting to the hospital more than 48 hours after ictus were excluded. Demographic data, Fisher group and Hunt and Hess grades were recorded on admission. Blood sampling was performed on the day of aneurysmal rupture and on post-hemorrhage days (PHD) 1,3,5,7 and 10. Magnetic resonance imaging was performed on PHD 14 in all surviving patients without a contraindication. Patients were treated according to a standard local protocol for the management of SAH [25]. Aneurysms were secured with either endovascular coiling or surgical clipping within 24 hours of presentation. Nimodipine was administered for 21 days. Anti-epileptic medications were administered for 7 days after ictus in the absence of seizures. Ventriculostomy catheters were placed in patients with a GCS <8 or with clinically significant hydrocephalus (n = 14).

Monitoring for cerebral vasospasm was routinely performed with daily trans-cranial Doppler (TCD) ultrasound, and post-operative angiography, while neurological evaluations evaluated the presence of DCI. TCD vasospasm was defined as a mean blood flow velocity greater than 125 cm/s in the anterior circulation or greater than 100 cm/s in the posterior circulation in addition to a Lindegaard ratio (mean middle cerebral artery flow velocity/mean cervical internal carotid artery flow velocity) of greater than 3. Normal saline solution and vasopressors were administered to improve neurological deficits in patients with suspected DCI. If symptoms persisted or were refractory to these measures, intra-arterial treatments were employed (e.g., intra-arterial nicardipine or balloon angioplasty). The degree of angiographic vasospasm was stratified by the percent luminal reduction in the most severely afflicted vessel [26]: 1–24% mild; 25–50% moderate; severe >50%. Any evidence of ischemia on diffusion-weighted imaging was recorded as radiographic infarction. The Glasgow Outcome Scale – Extended (GOSE) and modified Rankin Scale (mRS) were obtained at 6–9 months after discharge by telephone interview or mailed questionnaire. These procedures were performed by investigators blinded to the neurodegeneration biomarker findings.

### Quantification of neurodegeneration biomarkers

Eight markers were measured serially in CSF, five by a quantitative near-infrared Western blot technique and the remaining three by enzyme-linked immunofluorescence assays. These measurements were made by an investigator blinded to the clinical data. For the immunoblotting, the following antibodies were used: mouse anti-α-spectrin (MAB1622 at 1/1000; Millipore); mouse anti-14-3-3β (Santa Cruz #25276 at 1/1000); rabbit anti-14-3-3ζ (Santa Cruz SC-1019 at 1/1000); mouse anti-UCH-L1 (Abcam AB72911 at 1/1000); mouse anti-NSE (Abcam AB10169 at 1/1000). The secondary anti-rabbit and anti-mouse antibodies fluorescing in the near-infrared range were purchased from Licor, and used at 1/15,000 dilution. Images were captured using an Odyssey near-infrared system under conditions where serial dilutions of the protein load produced linear changes in band density.

### Sandwich enzyme-linked immunofluorescence assays

The enzyme-linked fluorescence immunoassays for calpain-cleaved α-spectrin N-terminal fragment and hypophosphorylated neurofilament H (NFHsmi35) use a mouse monoclonal capture antibody, a rabbit detecting antibody, and mouse-anti-rabbit IgG conjugated to horseradish peroxidase as the reporter probe. These immunoassays have been characterized and described in detail before [17]. Each assay was normalized using serial 2-fold dilutions of standard proteins: brain extract treated with activated calpain and a purified bovine spinal cord neurofilament preparation, respectively.

For the S100β sandwich ELISA, the S100β capture antibody (Abcam AB8334 at 1/10,000 dilution) was adsorbed onto Nunc maxisorp 96 well plates overnight at 4°C in 50 mM sodium carbonate buffer. After any remaining protein binding sites were blocked with 1% normal mouse serum in Tris-buffered saline supplemented with 0.05% Tween-20 (TTBS, pH 7.4), wells were washed four times in TTBS, then human CSF at 1% or varying concentrations of recombinant human S100β standards (Genway #288258) prepared in normal mouse serum in TTBS supplemented with 1 mM CaCl_2_ were added for 90 minutes at 22°C (100 µl per well). Wells were washed four times in TTBS, then the biotinylated anti-S100β detecting antibody was added (Abcam AB20327 at 1/5000 dilution in normal mouse serum/TTBS/1 mM CaCl_2_) for 1 hour at 22°C. This antibody was prepared by dialysis against phosphate-buffered saline (pH 7.4), followed by reaction with a 50-fold molar excess of N-hydroxysuccinimido-biotin as recommended by the manufacturer (Pierce EZ-Link biotin). After another four washes in TTBS, the streptavidin-horseradish peroxidase reporter probe was added (Invitrogen; 1/20,000 in 0.2% bovine serum albumin in TTBS/1 mM CaCl_2_) and incubated for 1 hour at 22°C. After four more washes in TTBS, the fluorogenic horseradish peroxidase substrate warmed to 37°C was added to each well. The substrate consisted of Amplex Ultrared (Invitrogen) prepared in 50 mM sodium citrate (pH 6.4) plus 0.003% hydrogen peroxide. Fluorescent product generated over 30 minutes at 37°C was measured using a plate-reading spectrofluorimeter (Packard Fluorocount) set for 485 nm excitation and 590 nm emission. Linear regression analysis of the S100β standards provided for normalization of CSF S100β levels across experiments.

For graphical representation of CSF levels of the eight biomarkers measured serially for up to 10 days for 14 aSAH cases, the marker concentrations were grouped into four categories: undetectable/low, medium, high, or very high. For the markers, 60% of the data points were classified as undetectable/low, 20% as medium, 10% as high, and 10% as very high. The peak units of each marker were coded as follows: CCSctf - 0–0.6 (blue), 0.61–2.0 (yellow), 2.01–5.0 (orange), 5.01-higher (red); NFHsmi35–0.3.0 (blue), 3.1–6.0 (yellow), 6.1–30 (orange), 30.1–higher (red); 14-3-3β - 0-3.0 (blue), 3.1–10 (yellow), 10.1–21 (orange), 21.1-higher (red); CCSntf – 0–4 (blue), 4.1–11 (yellow), 11.1–30 (orange), 30.1-higher (red); 14-3-3ζ - 0-3.9 (blue), 4.0–10.0 (yellow), 10.1–30 (orange), 30.1-higher (red); UCH-L1 – 0–3.0 (blue), 3.1–6.0 (yellow), 6.1–15.0 (orange), 15.1-higher (red); NSE – 0–3.0 (blue), 3.1–6 (yellow), 6.1–15.0 (orange), 15.1-higher (red); S100β - 0–40 (blue), 41–100 (yellow), 101–200 (orange), 201–higher (red).

For statistical analysis, the peak CSF level of each neurodegeneration biomarker was compared between groups dichotomized with respect to initial hemorrhage severity, radiological infarction, angiographic vasospasm, outcome at hospital discharge, and long-term outcome using two-tailed t-test.

## Results

### Severe aneurysmal subarachnoid hemorrhage patients enrolling in the neurodegeneration biomarker study

The demographics of the 25 severe aSAH patients enrolling in the current study are summarized in Table 1. Two of the patients withdrew from the study during the first 5 days of care and a third expired. Of the remaining 22 patients, 15 received a ventriculostomy and so provided serial CSF samples for neurodegeneration biomarker analyses. These patients are the focus of the remainder of the manuscript. For one patient (case 16), the ventricular drain did not remain patent and could not be replaced. Among the remaining 14 cases, 5 developed moderate to severe vasospasm beginning 5 days after aneurysm rupture as indicated by catheter angiography, and confirmed by increased TCD flow velocities and worsening of neurological symptoms. Twelve of the patients were evaluated for brain infarction two weeks after aneurysm rupture by diffusion-weighted MRI, and 6 had one or more foci of cerebral infarction. Eleven provided serial CSF samples and survived to hospital discharge. Based on their functional disabilities 2 returned home, 7 were discharged to an acute in-patient rehabilitation facility, and 2 were discharged to a skilled nursing facility. Among the 14 cases providing serial CSF samples, long-term outcome assessments by the GOSE and mRS were completed for 11 patients.

**Table 1:** Aneurysmal subarachnoid hemorrhage patient demographics.

Case #	Gender	HH/F Grade	Race	Age	Serial CSF
1	F	1/3	AA	45	No
2	F	4/3	C	37	No
3	F	5/3	C	54	Yes
4	F	2/3	C	49	No
5	F	½	H	35	No
6	M	1/3	AA	60	No
7	F	1/3	AA	53	No
8	M	1/3	C	51	No
9	F	2/3	C	51	Yes
10	F	2/3	C	51	Yes
11	F	2/3	C	26	No
12	F	5/4	C	57	Yes
13	M	5/4	H	63	Yes
14	M	Withdrawn	C	48	No
15	F	3/3	H	56	Yes
16	F	2/3	C	47	No
17	F	2/4	C	38	Yes
18	M	5/4	C	59	Yes
19	F	3/3	C	51	Yes
20	F	Withdrawn	C	67	No
21	F	4/4	AA	67	Yes
22	F	3/4	C	52	Yes
23	M	2/3	AA	50	Yes
24	M	4/3	C	51	Yes
25	F	5/4	AA	49	Yes

Abbreviations: HH/F – Hunt-Hess/Fisher scale; C – Caucasian; H – Hispanic; AA – African-American.

### The CSF levels of neurodegeneration biomarkers vary markedly over time and across aSAH patients

**Figure 1:**
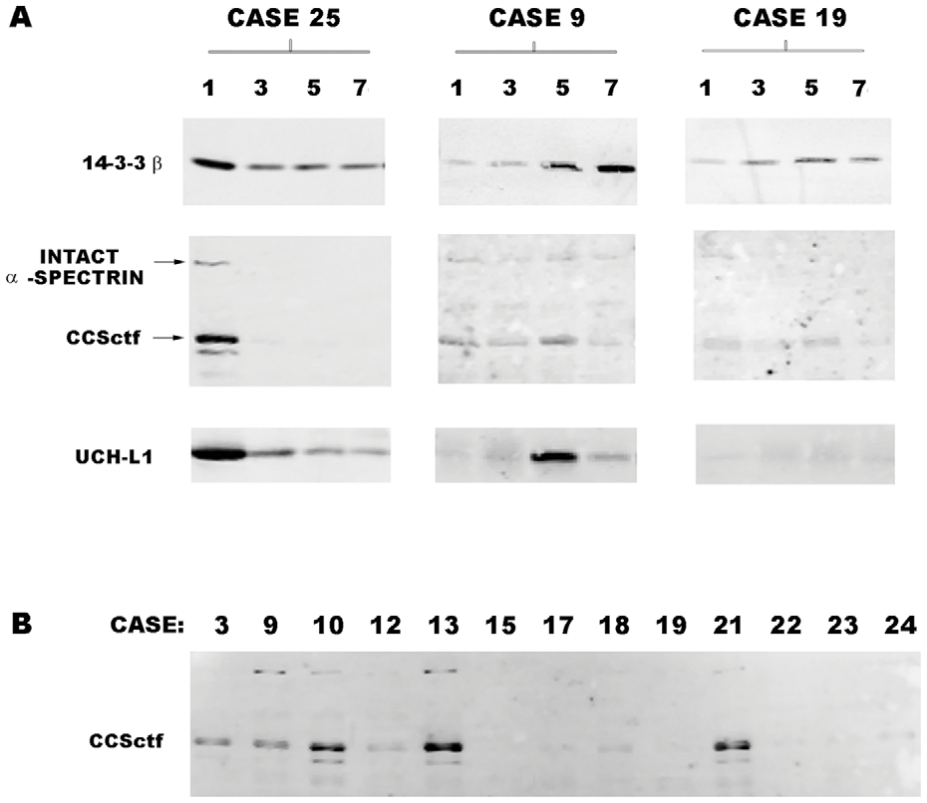
CSF changes in 3 neurodegeneration biomarkers after aneurysm rupture. (A) Western blot analyses of 14-3-3β, a calpain-cleaved α-spectrin COOH-terminal fragment (CCSctf), and UCH-L1 in 3 aSAH cases at the indicated times post-rupture (in days). Case 25 is representative of the subset of aSAH patients exhibiting large elevations in every neurodegeneration biomarker. Case 9, in contrast, shows a delayed increased in neurodegeneration biomarkers starting on day 5 after aneurysm rupture. Case 19 is representative of the subset of aSAH cases exhibiting consistently low or undetectable biomarker levels over the entire 10 day post-rupture period. (B) Western blot analysis of CCSctf levels 5 days post-rupture for 13 aSAH cases. Quantitative analysis (see Methods) showed that CSF levels of this biomarker vary by 100-fold across patients.

In addition to the neuron-enriched protein NSE and the astroglial-enriched protein S100β, which have been studied extensively as markers for acute brain damage, we measured serial CSF alterations in the neurodegeneration biomarkers 14-3-3ζ, 14-3-3β, calpain-cleaved α-spectrin N-terminal (CCSntf) and C-terminal fragments (CCSctf), ubiquitin C-terminal hydrolase L1 (UCH-L1), and a hypophosphorylated form of the high molecular weight neurofilament polypeptide (NFHsmi-35). These latter 6 proteins and NSE are expressed predominantly in neurons, are released upon neuronal degeneration [12]–[14] and, in the case of the two calpain derived proteolytic fragments, are generated preferentially in neurons undergoing calpain-driven necrosis [20], [22], [27]. All 7 of the neuronal protein markers along with S100β rose in a subset of aSAH patients during the post-hemorrhage period, and their levels varied markedly over time and across patients. Examples for 3 patients of the time- and patient-dependent CSF increases in the neurodegeneration biomarkers 14-3-3β, CCSctf, and UCH-L1 are shown in Figure 1. Case 25 exhibited large, rapid CSF elevations in these three proteins (Figure 1A), as well as all other members of the neurodegeneration biomarker panel. For case 9, on the other hand, neurodegeneration biomarkers were near the lower limit of detection to 3 days post-rupture, and rose either modestly (CCSctf) or markedly(14-3-3β, UCH-L1) beginning on day 5. Case 19 is an example in which CSF levels of every neurodegeneration biomarker were relatively low over the entire 7–10 day post-rupture period.

The peak CSF levels of the neurodegeneration biomarkers differed dramatically across aSAH patients. The CSF levels of one marker at a single time point post-rupture across 13 patients are illustrated in Figure 1B. At 5 days, CCSctf levels differed more than 100-fold between patients. The antibody recognizing CCSctf (∼150 kDa) also reacts with intact α-spectrin (∼250 kDa) and a smaller caspase-derived spectrin fragment (∼120 kDa) [28] but, unlike the calpain derivative, the levels of intact α-spectrin were low and variable across patients, and the caspase derivative was near the lower limit of detection in every case.

**Figure 2:**
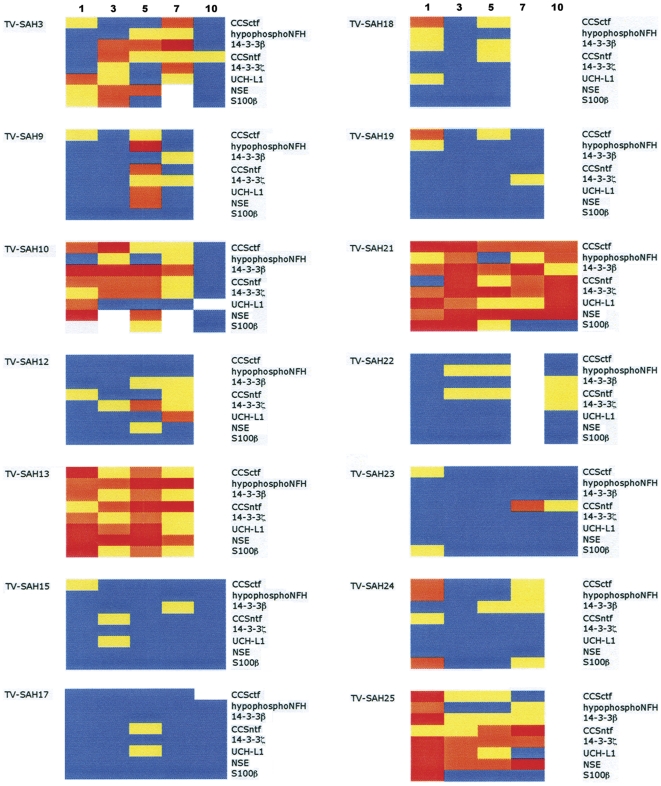
Summary of neurodegeneration biomarker increases up to 10 days after severe aSAH. The figure depicts CSF levels for 8 neurodegeneration biomarkers measured serially in a cohort of 14 severe aSAH cases. The levels of each marker have been divided into 4 categories as described in Methods , representing either low (blue), medium (yellow), high (orange), or very high (red).

The varying magnitudes and time courses for CSF elevations in the entire panel of 7 neurodegeneration biomarkers and S100β are summarized in Figure 2. To depict in a simplified fashion data for 8 markers measured in 14 patients at up to 5 time points, marker levels were categorized by color as either undetectable/low (blue), medium (yellow), high (orange), or very high (red). From this depiction, patients can be readily divided into 3 groups based on the neurodegeneration biomarker findings. Some patients demonstrated low or undetectable marker elevations over the entire 7–10 day post-hemorrhage period for most or all of the markers (cases 15, 17, 19, 22, 23). For other cases, most of the biomarkers increased only moderately (cases 12, 18, 24). Finally, in a third set of cases levels of the 7 neuron-enriched biomarkers (the entire panel less S100β) rose markedly (cases 3, 10, 13, 21, 25).

The 7 neurodegeneration biomarkers and S100β declined at different rates after reaching peak levels, providing evidence for their differential turnover in the CSF compartment. For example, CCSctf has a short half-life in human CSF as evidenced by its ∼80-fold decline in case 25 from day 1 to 3. Although steady-state levels of these markers are dependent not only on their rates of clearance but also their continuing rates of production, and so a precise measure of their half-lives is not feasible from steady-state data alone, it can be estimated that the CSF half-life of CCSctf is less than 5 hours. Among the remaining markers, the half-life of S100β was also relatively short, as has reported previously [29], whereas 14-3-3 β and ζ forms, UCH-L1, NFHsmi-35, and CCSntf had slower rates of decay after reaching peak levels.

### Evidence that a set of 7 neurodegeneration biomarkers predicts cerebral vasospasm, infarction, and functional outcome

To begin evaluating the prognostic potential of this panel of neurodegeneration biomarkers for aSAH, we compared the time course and magnitude of marker elevations with the incidence and severity of cerebral vasospasm, infarction, and brain dysfunction at hospital discharge and long-term follow-up. Of the five confirmed cases of moderate or severe cerebral vasospasm (cases 3, 9, 10, 21, and 22), four showed large elevations in at least 6 neurodegeneration biomarkers within 7 days post-hemorrhage. Only case 22 experienced a moderate angiographic vasospasm that was not accompanied by large elevations in the neurodegeneration biomarker panel. Strikingly, case 22 also was the only example of a moderate/severe vasospasm that was not accompanied by brain infarction visible with diffusion-weighed imaging. In some patients, biomarker changes were related temporally to the onset of vasospasm. A clear example is provided by case 9, in which a marked increase in blood flow velocities in the right middle and anterior cerebral arteries started on day 6, the time when the Lindegaard ratio first indicated vasospasm (confirmed by catheter angiography). CSF levels of 7 of the 8 biomarkers were very low in this case for up to 3 days post-hemorrhage, but thereafter showed large spikes in 7 of the markers, starting at day 5. Thus, neurodegeneration biomarker elevations occurred in this case prior to the detection of cerebral vasospasm by ultrasonographic and angiographic measures.

**Figure 3:**
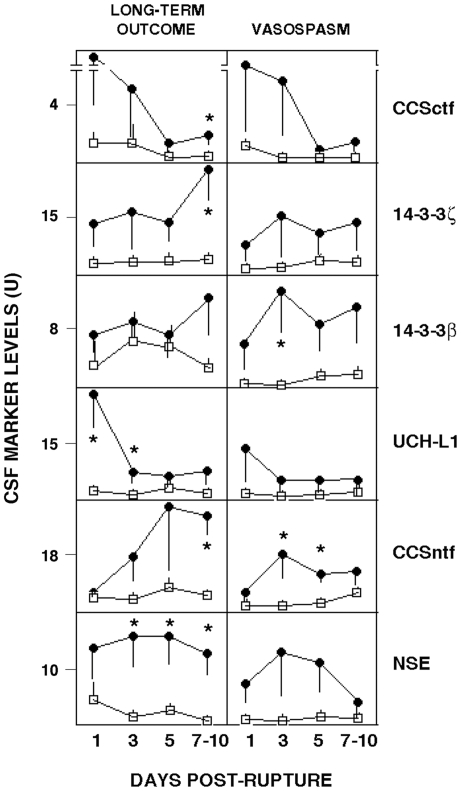
Time courses for 6 CSF neurodegeneration biomarkers in relation to functional outcome and the severity of cerebral vasospasm. Long-term outcome was dichotomized to either poor (closed circles: GOS-E  = 1–4; mRS  = 4–6) or good (open boxes: GOS-E  = 5–8; mRS  = 1–3), while angiographic vasospasm was categorized as either moderate/severe (closed circles) or absent/mild (open boxes) as described in Methods. Mean CSF levels of each neurodegeneration biomarker at the indicated times post-aneurysm rupture (in days) are shown with the standard error of the mean. Statistically significant between-group differences (p<0.05) are identified by asterisk. In cases for which CSF samples were available at both 7 and 10 days post-rupture, the higher marker level was considered.

The temporal patterns of CSF changes in 6 neurodegeneration biomarkers are shown in Figure 3 in relation to dichotomized assessments of cerebral vasospasm and long-term brain functional outcome. In the group of patients experiencing angiographic vasospasm that was moderate or severe, levels of all 6 neurodegeneration biomarkers were elevated by from 3- to 10-fold between days 1–5 post-rupture. The increase in 14-3-3β was significant at day 3, whereas the rise in CCSntf was significant at days 3 and 5. These early biomarker increases are noteworthy in that moderate to severe vasospasms typically became apparent from 5–14 days post-rupture, as evidenced by vessel narrowing visualized by angiography, and supported by increased anterior blood flow velocities and worsening neurological signs. Neurodegeneration biomarker peak elevations also were related to long-term outcome, as aSAH patients with poor long-term outcomes (GOS-E 1-4/mRS4-6) had consistently and markedly higher CSF levels of all 6 neurodegeneration biomarkers (up to 20-fold increases) over the entire 10 day post-hemorrhage period. Statistically significant increases were observed at 7–10 days for the two calpain-derived α-spectrin derivatives CCSctf and CCSntf, 14-3-3ζ, and NSE, while CSF levels of UCH-L1 and NSE were significantly related to long-term outcome at earlier post-hemorrhage times.

The data comparing peak levels of the 8 biomarkers to poor functional outcome and pathophysiological processes contributing to it are summarized in Tables 2, 3, 4, 5, 6. The peak neurodegeneration biomarker levels from 5–10 days after aneurysm rupture were related to the initial severity of the SAH as determined by the Hunt-Hess grade. Peak marker increases were up to 6-fold higher in patients presenting with Hunt-Hess grades of 4 or 5 compared with grades of 2 or 3 (Table 2). In addition, the peak CSF concentrations of all 8 markers were from 4 to 20 fold higher in cases with focal infarcts detected by diffusion-weighed MRI, and moderate-to-severe vasospasms detected by angiography and supported by ultrasonography. The peak CSF level of CCSctf from 3–10 days post-hemorrhage correlated significantly with the incidence of infarction (Table 3). CSF 14-3-3β, CCSntf, and NSE also were early predictors of the subsequent development of vasospasm, with their peak levels within the first 3 days correlating significantly with angiographic vasospasm that was moderate or severe (Table 4).

**Table 2:** Relationship between peak CSF neurodegeneration biomarker levels and hemorrhage severity.

Grade	CCSctf	NFHsmi35	14-3-3β	14-3-3ζ	UCH-L1	CCSntf	NSE	S100β
2–3	0.5 +/- 0.2	12.9 +/- 11.4	7.7 +/- 4.5	5.3 +/- 1.2	2.8 +/- 1.9	8.6 +/- 2.0	2.3 +/- 1.2	21 +/- 11
4–5	1.7 +/- 0.4	17.1 +/- 12.8	13.8 +/- 5.3	16.5 +/- 6.0	7.0 +/- 2.4	33.1 +/- 16.7	13.1 +/- 4.4	39 +/- 14
P value	**0.03**	0.81	0.39	0.09	0.21	0.17	**0.04**	0.35

The mean (+/- S.E.M.) peak level of each biomarker from 3–10 days post-rupture is depicted in relation to the initial severity of the aneurysm dichotomized according to the Hunt-Hess grade.

**Table 3:** Relationship between peak CSF neurodegeneration biomarker levels and neuroradiologically confirmed cerebral infarction.

Infarction	CCSctf	NFHsmi35	14-3-3β	14-3-3ζ	UCH-L1	CCSntf	NSE	S100β
Yes	1.8 +/- 0.4	17.3 +/- 12.7	18.9 +/- 6.9	13.7 +/- 6.8	6.3 +/- 2.8	14.5 +/- 5.0	10.0 +/- 4.5	40.1 +/- 12.5
No	0.3 +/- 0.1	1.2 +/- 0.8	3.3 +/- 0.5	3.6 +/- 0.8	0.6 +/- 0.1	4.8 +/- 1.1	0.5 +/- 0.2	8.4 +/- 2.1
P value	**0.03**	0.34	0.11	0.27	0.14	0.16	0.13	0.08

The mean (+/- S.E.M.) peak level of each biomarker from 3–10 days post-rupture is depicted in relation to infarction detectable by diffusion weighted MRI.

**Table 4:** Relationship between early CSF neurodegeneration biomarker increases and the severity of cerebral vasospasm.

Vasospasm	CCSctf	NFHsmi35	14-3-3β	14-3-3ζ	UCH-L1	CCSntf	NSE	S100β
No/Mild	1.6 +/- 0.5	4.7 +/- 2.5	1.3 +/- 0.4	2.6 +/- 0.6	2.0 +/- 0.6	3.2 +/- 0.9	1.1 +/- 0.3	50 +/- 22
Mod/Severe	10.7 +/- 8.4	3.7 +/- 0.8	20.6 +/- 9.7	17.3 +/- 12.7	12.9 +/- 8.9	17.9 +/- 6.9	14.7 +/- 6.8	350 +/- 285
P value	0.22	0.77	**0.04**	0.19	0.17	**0.03**	**0.04**	0.24

The mean (+/- S.E.M.) peak level of each biomarker within 3 days of aneurysm rupture is depicted in relation to dichotomized severity of angiographic vasospasm.

**Table 5:** Relationship between peak CSF neurodegeneration biomarker levels and outcome at hospital discharge.

Outcome	CCSctf	NFHsmi35	14-3-3β	14-3-3ζ	UCH-L1	CCSntf	NSE	S100β
Dead, Nursing	2.3 +/- 0.6	27.1 +/- 22.1	20.9 +/- 7.7	24.7 +/- 8.4	9.8 +/- 3.5	53.8 +/- 25.5	21.3 +/- 4.0	54 +/- 22
Rehab/Home	0.7 +/- 0.2	10.9 +/- 8.8	6.9 +/- 3.5	4.8 +/- 1.0	2.4 +/- 1.5	7.9 +/- 1.6	2.1 +/- 0.9	22 +/- 9
P value	**0.005**	0.42	0.08	**0.004**	**0.04**	**0.02**	**0.0001**	0.13

The mean (+/- S.E.M.) peak level of each biomarker from 3–10 days post-rupture is depicted in relation to dichotomized discharge outcome.

**Table 6:** Relationship between peak CSF neurodegeneration biomarker levels and long-term outcome.

Outcome	CCSctf	NFHsmi35	14-3-3β	14-3-3ζ	UCH-L1	CCSntf	NSE	S100β
GOSE 5-8	0.6 +/- 0.2	17.8 +/- 15.8	9.4 +/- 6.3	4.6 +/- 2.1	3.6 +/- 2.7	9.0 +/- 2.5	2.9 +/- 1.6	27 +/- 15
GOSE 1-4	2.0 +/- 0.5	22.3 +/- 17.8	17.5 +/- 6.8	20.5 +/- 7.7	8.2 +/- 3.2	44.3 +/- 21.9	17.3 +/- 5.0	127 +/- 59
P value	**0.04**	0.86	0.41	0.08	0.29	0.15	**0.03**	0.14

The mean (+/- S.E.M.) peak level of each biomarker from 3–10 days post-rupture is depicted in relation to long-term outcome dichotomized according to the Glasgow Outcome Scale - Extended.

In addition to relating to the radiologic, angiographic, and ultrasonographic evidence of cerebral infarction and vasospasm, peak levels of all 8 of the markers were related strongly to the severity of brain dysfunction assessed at the time of hospital discharge. Case 12 did not show evidence of vasospasm or brain infarction, but had a second aneurysm rupture on day 10 that proved fatal. This case was excluded from comparative analyses of neurodegeneration biomarker elevations in relation to outcomes. For the remaining cases, those that either expired or were discharged to a nursing facility had higher CSF levels of all 8 biomarkers ranging from 3 to 10-fold when compared with cases discharged either to home or an in-patient rehabilitation facility. The peak elevations in CCSctf, 14-3-3ζ, UCH-L1, CCSntf, and NSE were correlated significantly with poor discharge outcome (Table 5).

Finally, the panel of neurodegeneration biomarkers correlated with poor functional outcome at 6-9 months as assessed by the GOSE and mRS. For 6 of the 7 neurodegeneration markers, peak marker levels measured within 10 days of aneurysm rupture were from 2 to 6-fold higher in patients with poor long-term functional outcomes. Peak CSF levels of both CCSctf and NSE measured from 3-10 days post-rupture differed significantly between GOSE and mRS dichotomized to good (GOSE 5-8/mRS 1-3) or poor (GOSE 1-4/mRS 4-6) brain functional outcome (Table 6).

## Discussion

In this report we describe a panel of neurodegeneration biomarkers that rise up to 100-fold in CSF following severe aSAH and collectively may be early predictors of pathophysiological complications and poor functional outcome. In addition to the widely studied NSE and S100β, 6 additional neuron-enriched proteins that are released from degenerating neurons increase within a week of aneurysm rupture. Peak levels of a group of 6 markers (CCSctf, CCSntf, 14-3-3β, 14-3-3ζ, UCH-L1, and NSE) correlate significantly with moderate-to-severe cerebral vasospasm, focal infarction, and lasting brain dysfunction, whereas no single marker correlates with every measure. These findings identify a biomarker panel meriting consideration as clinically useful prognostic measures of ischemic brain damage.

A major challenge in aSAH research has been to establish biochemical markers capable of predicting the development of DCI and lasting brain dysfunction. Such markers could serve as surrogate endpoints for controlled clinical research studies of the comparative efficacy of treatment interventions designed to normalize physiology, reduce DCI, and improve outcome. A handful of proteins have been studied in this regard, including the neuron-enriched NSE and neurofilament L, along with the glial-enriched S100β, glial fibrillary acidic protein, and myelin basic protein [7]-[13], but unfortunately none has proven a reliable surrogate measure for the vasospasm, DCI, or poor functional outcome of aSAH. Recently, we and others identified additional proteins released from degenerating neurons whose efflux from the injured brain could be potential surrogate measures for ischemic brain damage, including two calpain-derived proteolytic fragments of the actin-binding cytoskeletal protein α-spectrin, the ubiquitin ligase UCH-L1, three distinct phosphoforms of the high molecular weight neurofilament subunit NFH, and the β and ζ isoforms of 14-3-3 protein [9]–[12], [14]–[18]. We report that no single marker correlates significantly with every measure of complications and outcomes, consistent with prior studies in aSAH. Instead, we present evidence from collective analyses of a new panel of 7 neurodegeneration biomarkers along with the astroglial-derived S100β that combinations of 6 of the markers predict moderate to severe cerebral vasospasm (14-3-3β, CCSntf, NSE; Table 4), focal ischemic brain damage (CCSctf; Table 3), and brain dysfunction at hospital discharge (CCSctf, 14-3-3ζ, UCH-L1, CCSntf, NSE; Table 5) and long-term follow-up (CCSctf, NSE; Table 6). These data support the hypothesis that a panel of neurodegeneration biomarkers has far more discriminative power than any single marker alone to distinguish pathophysiological complications and brain damage following aSAH, and potentially other acute neurodegenerative disorders as well. The significant correlations between neurodegeneration biomarker increases, long-term brain dysfunction, and the cerebral vasospasm and infarction that contribute to the lasting dysfunction, coupled with prior reports linking these markers to multiple forms of ischemia- and trauma-induced acute brain damage [11], [12], [14]–[19], suggest that the marker panel could serve as a surrogate endpoint for therapeutic response in controlled clinical trials. The responsiveness of the neurodegeneration biomarkers to interventions that reduce pathophysiological complications and improve functional outcomes will require further study.

Not only are delayed elevations in the panel of neurodegeneration biomarkers related to complications and outcomes after severe aSAH, but their early peak levels measured within 3 days of aneurysm rupture may predict the subsequent development of moderate to severe cerebral vasospasm. Although the initial assessment of aneurysm severity by the Fisher, Hunt-Hess, or WFNS grade has been associated with the subsequent risk for cerebral vasospasm and DCI [30], [31], some studies report the relationship is weak [26], [32]. The early biomarker-based detection at a preclinical stage of aSAH-induced cerebral vasospasm might have implications for preventive treatments of vasospasm by improving its early detection and widening the therapeutic window beyond that achievable from clinical, ultrasonographic, and angiographic information. A driving force for the rapid rise in CSF neurodegeneration biomarkers could be early brain injury that shortly follows aneurysm rupture, and is thought to be a strong determinant of subsequent mortality and morbidity [33]. Consistent with the importance of primary injury mechanisms, the stratification of hemorrhage severity by the Hunt-Hess grade was significantly related to the delayed CSF elevations in the neurodegeneration biomarkers CCSctf and NSE occurring 7–10 days later (Table 2).

The present single-site clinical study is limited by the small sample size, but provides preliminary evidence with internal, external, and mechanistic support that a panel of neurodegeneration biomarkers might be prognostic measures for severe aSAH. Rather than simply relying on only one or two markers, we describe that 6 proteins released by degenerating neurons rise markedly in association with lasting brain dysfunction. This association is strengthened further by the relationships between the neurodegeneration biomarker elevations and pathophysiological processes (cerebral vasospasm and infarction) contributing to long-term dysfunction. The link between these neurodegeneration biomarker increases and acute brain damage is not limited to SAH, since several members of the marker panel are also associated with other forms of ischemia- and trauma-induced brain injury [14]–[21]. Two members of the marker panel, the N- and C-terminal derivatives of α-spectrin, are mechanism-based markers, in that they are derived from proteolytic processing by the calpain family of cysteine proteases that play an important role in neuronal necrosis [20], [34]. Using a single antibody reactive not only with a calpain- but also a caspase-derived α-spectrin fragment, we provide CSF biomarker evidence that calpain-driven necrosis predominates over caspase-mediated apoptosis as a neurodegenerative mechanism in the human brain following severe aSAH (Figure 1). These data are consistent with histopathological and biomarker reports of the prevalence of calpain-driven necrosis in experimental models of ischemic and traumatic brain injury, as well as prior studies of these mechanism-based neurodegeneration biomarkers in humans following surgically-induced circulation arrest or severe TBI 15-18,23,.

In light of the small sample size evaluated thus far, the application of neurodegeneration biomarker elevations as early predictors of the subsequent development of cerebral vasospasm, infarction, and long-term brain dysfunction in SAH will require further study in larger patient cohorts. In addition to early prognosis, there are several additional features of aSAH that may be addressed by a panel of neurodegeneration biomarkers in larger multi-center clinical studies. Among the panel members are protein components of both neurons and astroglia, and the neuron-enriched proteins differ in both their brain regional and subcellular localizations. For example, various phosphoforms of NFH have been proposed as selective markers for axonal vulnerability following brain injury [35]. Furthermore, although all members of the neurodegeneration biomarker panel are widely expressed across the neuraxis, their expression levels are not uniform across all brain regions and neuronal types. Consequently, it may be possible to use the current or a more extensive next-generation panel of biomarkers that include proteins with restricted expression across brain regions, cell populations, and subcellular compartments to inform on a per-patient basis the anatomical localization of acute brain damage, the susceptibility of distinct cell types, and the contribution of axonal injury.

In conclusion, this study provides evidence that a panel of 6 neurodegeneration biomarkers might predict cerebral vasospasm, infarction, and lasting brain dysfunction following severe aSAH. The findings suggest that a panel of neurodegeneration biomarkers could have enhanced discriminative power over any single marker for predicting pathophysiological complications and the ensuing acute brain damage and lasting brain dysfunction. Such a neurodegeneration biomarker panel could be valuable as surrogate endpoints for controlled clinical evaluation of treatment interventions designed to mitigate ischemic brain damage and improve functional outcome. More extensive clinical study of the neurodegeneration biomarkers described here in well-monitored patients will be required to define their diagnostic, prognostic, mechanistic, and experimental therapeutic applications in aSAH and other forms of acute brain damage.
